# Supplementary material for: Iron Deficiency in Cystic Fibrosis: A Cross-Sectional Single-Centre Study in a Referral Adult Centre
Source: Nutrients. 2022 Feb 5;14(3):673. doi: 10.3390/nu14030673 (PMC8838796; doi:10.3390/nu14030673)
Supplement: Supplementary file 1 [file nutrients-1577545-supplementary.pdf › nutrients-1577545-supplementary.pdf]

mut1	mut2
DF508	DF508
DF508	DF508
DF508	DF508
DF508	DF508
DF508	DF508
DF508	DF508
DF508	DF508
DF508	DF508
DF508	DF508
DF508	DF508
DF508	DF508
DF508	DF508
DF508	DF508
DF508	DF508
DF508	DF508
DF508	DF508
DF508	DF508
DF508	DF508
DF508	DF508
DF508	DF508
DF508	DF508
DF508	DF508
DF508	DF508
DF508	DF508
DF508	DF508
DF508	DF508
DF508	DF508
DF508	DF508
DF508	DF508
DF508	DF508
DF508	DF508
DF508	DF508
DF508	DF508
DF508	DF508
DF508	DF508
DF508	DF508
DF508	DF508
DF508	DF508
DF508	DF508
DF508	DF508
DF508	DF508
DF508	DF508
DF508	DF508
DF508	DF508
DF508	DF508
DF508	DF508
DF508	DF508
DF508	DF508
DF508	DF508

mut1	mut2
DF508	(TG)12T5
DF508	1717 - 1G>A
DF508	1717 - 1G>A
DF508	811+1,6kbA>G
DF508	2043delG
DF508	2183 AA>G
DF508	2183 AA>G
DF508	2711 del T
DF508	2711 del T
DF508	2711 del T
DF508	2711 del T
DF508	2789 + 5G>A
DF508	2789 + 5G>A
DF508	2789 + 5G>A
DF508	3659 del C
DF508	849 + 10 kb C>T
DF508	849 + 10 kb C>T
DF508	849 + 10 kb C>T
DF508	621+1G>T
DF508	711 + 1 G>T
DF508	C2657 +GT>A
DF508	CFTR dele2,3
DF508	CFTRdele2-10
DF508	D1152H
DF508	D1152H
DF508	D1152H
DF508	D1152H
DF508	E60X
DF508	E60X
DF508	G542X
DF508	G542X
DF508	G542X
DF508	G542X
DF508	G542X
DF508	G542X
DF508	G542X
DF508	G628R (G>A)
DF508	G85E
DF508	G85E
DF508	H1054D
DF508	I86M
DF508	indéterminé
DF508	IVS5 + 5G>A
DF508	L137R
DF508	L206W
DF508	L32P 227T>C
DF508	N1303K
DF508	N1303K
DF508	N1303K

DF508	DF508
DF508	DF508
DF508	DF508
DF508	DF508
DF508	DF508
DF508	DF508
DF508	DF508
DF508	DF508
DF508	DF508
DF508	DF508
DF508	DF508
DF508	DF508
DF508	DF508
DF508	DF508
DF508	DF508
DF508	DF508
DF508	DF508
DF508	DF508
DF508	DF508
DF508	DF508
DF508	DF508
DF508	DF508
DF508	DF508
DF508	DF508
DF508	DF508
DF508	DF508
DF508	DF508
DF508	DF508
DF508	DF508
DF508	DF508
DF508	DF508
DF508	DF508

DF508	N1303K
DF508	R1162X
DF508	R1162X
DF508	R117H
DF508	R347P
DF508	R553X
DF508	R553X
DF508	R553X
DF508	W1282X
DF508	W401
DF508	Y1092X
DF508	Y122X

mut1	mut2
1525 - 1G>A	1525 - 1G>A
2347 del G	K52X
2711 del T	2711 del T
3272-26A>G	3272-26A>G
5T	5T
5T	indéterminé
5T	indéterminé
711 + 1G>T	G576A
CFTR dele 2,3	H199R
E585X	R1066C
G139D	2183 AA>G
G542X	R347P
G542X	TR dele 14b - 17b
G542X	R347P
G542X	G628R (G>A)
H199R	N1303K
L227R	L227R
N1303K	2789 + 5G>A
N1303K	DF508
R334W	N1101K
R334W	M1101K
R553X	2789 + 5G>A
W1282X	1677delTA
